# The data on exploratory factor structure of [pre-service] teacher beliefs about educational research scale

**DOI:** 10.1016/j.dib.2020.106578

**Published:** 2020-11-25

**Authors:** Yoppy Wahyu Purnomo, Irfan Wahyu Prananto, Elin Driana, Kiki Saparanti, Ishaq Nuriadin, Siti Noor Ismail

**Affiliations:** a Universitas Negeri Yogyakarta, Indonesia; b Universitas Muhammadiyah Prof. Dr. HAMKA, Indonesia; c Universiti Utara Malaysia, Malaysia

**Keywords:** Teacher beliefs, Educational research, Exploratory factor analysis, Pre-service teachers

## Abstract

This article presents data on factors that depict [pre-service] teacher beliefs about educational research. A cross-sectional survey was used to collect the data that involved 352 final-year students working on their undergraduate theses. The students were registered in the faculty of teacher training and education in one of the private universities with an A (excellent) accreditation in Jakarta, Indonesia. The participants were selected conveniently. Exploratory factor analysis was performed to reveal the factors comprised in the data. 49 out of 72 items formed five factors: negative views about educational research, positive views about educational research, knowledge about educational research, open-mindedness, and accessibility.

## Specifications Table

**Table:** 

Subject	Education
Specific subject area	Pre-service teacher, teacher education, teacher beliefs
Type of data	Table
How data were acquired	A cross-sectional survey method was employed to collect the data. The data were derived from a questionnaire. The questionnaire is provided as a supplementary file in this paper.
Data format	Raw Analyzed
Parameters for data collection	The cross-sectional survey was adopted to target pre-service teachers who were administratively enrolled as students of the 8th semester or more.
Description of data collection	A total of 352 pre-service teachers completed this survey, who were selected using a convenience sampling method. The sampling was done at a private university in Jakarta, Indonesia. After the screening process, 352 data were evaluated using the Exploratory Factor Analysis (EFA).
Data source location	East Jakarta, DKI Jakarta, Indonesia
Data accessibility	The data are available in Mendeley Data: https://data.mendeley.com/datasets/fm8hvpd9w7/1

## Value of the Data

• One of the factors of gaps between educational research and practices is teacher beliefs about educational research. However, a tool for identifying this problem is still rare. This data describes an exploratory analysis of the factors included in teacher beliefs about educational research.
• The data can be useful for researchers who are interested in teacher education and professional teacher development.
• It is expected that the data can be used more effectively in further research to collect data on the factors that influence research-based teacher practice.
• The data serve as initial attempts to validate the five-factor constructs of teacher beliefs about educational research.
• The data can be compared with future research using the questionnaire with groups of in-service teachers.

## 1 Data Description

The data were derived from 72 questionnaire items for measuring teacher beliefs about educational research. The questionnaire is provided as a supplementary file in this paper or mendeley data (see http://dx.doi.org/10.17632/fm8hvpd9w7.1). Data in this article consists of two parts. The first part (see Table 1) is data that comprise factor loadings, mean of each item, and total variance that contribute to each factor. The data also comprises the degree of internal consistency and corrected item-total correlation (CITC). The second part (see Table 2) includes the mean of each factor, the standard deviation, and the range. The data also comprises correlations among factors.

**Table 1:** Factor loadings, CITC, variance explained, and internal consistency.

Statements	Component					M	SD	CITC
	**1**	**2**	**3**	**4**	**5**			
44. Teachers will not have enough time to manage between teaching and learning activities and research activities.	**0.698**	0.074	−0.150	0.012	0.002	3.946	1.174	0.581
50. Educational research was carried out because of administrative encouragement (graduation; rank).	**0.693**	−0.149	−0.023	−0.043	−0.234	4.242	1.236	0.570
37. Educational research is a matter of researchers' personal interest.	**0.658**	0.074	−0.125	−0.124	−0.188	3.815	1.322	0.512
28. Educational research is only useful for administrative purposes (graduation; rank).	**0.620**	−0.247	0.044	0.004	−0.144	3.432	1.390	0.532
54. Research is carried out because of encouragement from rules and policies.	**0.617**	0.070	0.015	0.016	−0.244	4.489	0.970	0.476
47. Research takes time away from other teacher's responsibilities.	**0.612**	−0.062	0.080	0.041	0.120	3.824	1.200	0.575
35. Educational research results are not convincing enough to be recognized for accuracy.	**0.580**	−0.067	0.059	−0.057	−0.006	3.832	1.230	0.524
49. My colleagues and I often wonder why teachers have to do research.	**0.562**	0.057	−0.063	0.021	−0.115	4.367	1.176	0.435
41. There is no problem even though there is no research.	**0.549**	−0.056	0.028	−0.105	−0.096	2.923	1.293	0.444
23. Educational research results do not have a significant impact on teaching practice.	**0.536**	−0.207	0.013	−0.050	0.175	3.500	1.265	0.528
52. I have not felt the impact of research on the educational field until now.	**0.523**	0.016	−0.177	−0.023	0.094	3.631	1.256	0.447
39. Learning theories are often not relevant to the reality of the educational settings.	**0.509**	−0.036	0.151	0.086	0.067	4.415	1.101	0.472
26. Educational research is often out of sync with the policies implemented.	**0.506**	0.124	−0.074	0.101	0.196	4.358	0.944	0.453
21. Educational research results and suggestions are often not relevant to the problems encountered in class.	**0.483**	−0.005	0.048	0.138	0.220	4.0577	1.061	0.479
19. Teaching practices that are based on research results and suggestions are difficult because of time constraints.	**0.465**	0.158	0.022	0.116	0.203	4.375	0.962	0.433
31. Educational research suggestions are difficult to apply in the form of real practice.	**0.422**	0.010	0.155	−0.034	0.269	4.227	1.083	0.455
20. Educational research can develop teacher's knowledge for teaching.	−0.057	**0.651**	−0.042	−0.062	0.014	5.119	0.665	0.522
36. Educational research develops the ability to make careful planning.	0.052	**0.642**	0.019	−0.077	0.032	5.028	0.574	0.519
33. Educational research is needed as a solution to overcome educational problems.	−0.031	**0.621**	−0.013	0.013	0.068	5.136	0.612	0.534
29. Educational research develops my logical thinking ability.	−0.079	**0.603**	0.064	−0.132	0.006	5.114	0.593	0.491
25. Educational research is needed to formulate educational policies.	0.030	**0.597**	−0.074	0.005	0.003	4.943	0.624	0.463
30. Educational research trains teachers to adapt to various learning conditions and situations.	−0.059	**0.589**	0.064	−0.019	−0.062	5.125	0.614	0.534
24. Educational research helps improve student learning.	0.081	**0.565**	−0.081	0.061	−0.314	4.966	0.727	0.503
32. Educational research is needed to evaluate what has been taught, to what extent, and what is needed next.	−0.049	**0.551**	−0.188	0.099	0.051	5.139	0.534	0.416
22. Educational research results and suggestions add alternative solutions to problems faced in class.	0.005	**0.551**	−0.101	0.082	−0.132	4.986	0.597	0.465
34. Educational research results have theoretical and/or practical views.	0.046	**0.519**	0.058	−0.070	−0.132	4.992	0.726	0.418
38. Educational research develops the ability of teachers to manage classrooms and interact with students.	−0.008	**0.500**	0.110	0.079	−0.035	5.046	0.678	0.481
42. Educational research helps to recognize student characteristics.	0.023	**0.498**	0.189	−0.002	−0.025	5.051	0.690	0.448
18. Educational research leads to teacher instruction improvement.	−0.179	**0.478**	0.205	−0.023	0.022	5.023	0.712	0.453
60. It is important to publish educational research results.	−0.159	−0.201	**0.790**	−0.038	−0.124	4.688	0.981	0.343
61. Research and publications complement each other.	−0.057	−0.112	**0.696**	−0.014	−0.162	4.784	0.843	0.429
62. The research method depends on the research purpose.	0.018	0.167	**0.520**	0.028	0.051	5.034	0.613	0.404
64. Problem statements can be derived from a review of previous research results.	0.175	0.105	**0.485**	0.013	−0.032	4.591	0.986	0.440
68. Good references take precedence over research results.	0.036	0.188	**0.483**	−0.018	0.109	4.906	0.739	0.402
58. A good title is able to reflect the contents of the research.	−0.119	0.119	**0.481**	0.026	0.143	5.017	0.813	0.315
70. Research results must be used as references more than textbooks.	0.217	0.053	**0.371**	−0.184	−0.029	4.460	0.966	0.442
66. Research is conducted to test the theories that have been formulated.	0.087	0.218	**0.328**	0.195	−0.009	4.901	0.683	0.347
1. The teacher must accept a variety of possible opinions before deciding on something.	−0.008	−0.039	−0.102	**0.708**	0.091	5.190	0.778	0.476
2. Teachers must be good listeners.	−0.096	−0.283	0.179	**0.675**	−0.060	5.531	0.787	0.379
6. Teachers must consider the input from others, even if it contradicts their personal opinions.	0.022	0.110	−0.073	**0.635**	0.056	5.165	0.684	0.453
7. Teachers must be able to accept something different from what they believe is best.	0.165	−0.067	−0.051	**0.614**	−0.051	4.903	0.706	0.493
4. Many advantages are gained when discussing problems faced in class with peers.	−0.069	0.092	−0.078	**0.586**	−0.069	5.130	0.679	0.395
9. It is important to try to implement a different approach from what has been done so far.	−0.042	0.141	0.030	**0.445**	−0.067	4.994	0.675	0.355
10. There are many alternatives that can be done to solve problems in the classroom.	0.027	0.165	0.131	**0.376**	−0.178	5.307	0.547	0.339
12. Articles or other forms of research reports are easy to access and/or find in online media.	0.273	0.098	0.129	0.101	**−0.675**	4.753	0.911	0.252
11. Articles or other forms of research reports are difficult to obtain.	0.181	0.096	−0.009	−0.186	**0.648**	3.477	1.217	0.458
14. Articles or other forms of research reports require expensive fees to get them.	0.337	−0.040	0.010	0.041	**0.428**	3.301	1.130	0.412
15. Communicating about educational research with experts/researchers/lecturer is easy.	0.073	0.235	0.036	−0.178	**−0.410**	4.125	1.068	0.178
17. Limited funds are a problem in obtaining literature.	0.285	0.034	0.112	0.027	**0.346**	4.009	1.161	0.313
**Explained variance**	12.912	11.756	4.807	3.927	3.797			
**Eigenvalues**	6.327	5.760	2.355	1.924	1.860			
**Cronbach's Alpha**	0.864	0.833	0.683	0.703	0.561

**Table 2:** Mean, deviation standard, range, and correlation analyses.

Factors	N	Mean	SD	Range	1	2	3	4	5
1. Negative Views	16	3.964	0.437	2.923–4.489	1.000	−0.042	0.152	−0.048	0.220
2. Positive Views	13	5.051	0.070	4.943–5.139	−0.042	1.000	0.309	0.353	−0.003
3. Knowledge about research	8	4.798	0.205	4.460–5.034	0.152	0.309	1.000	0.117	0.088
4. Open-mindedness	7	5.175	0.205	4.903–5.531	−0.048	0.353	0.117	1.000	0.003
5. Accessibility	5	3.182	0.662	2.247–4.009	0.220	−0.003	0.088	0.003	1.000

Table 1 shows that there are five factors in the questionnaire data on teacher beliefs about educational research, namely (1) negative views on educational research, (2) positive views about educational research, (3) knowledge about educational research, (4) open-mindedness and (5) accessibility. The first factor consists of 16 items with a degree of internal consistency of 0.864, the second factor consists of 13 items with a degree of internal consistency of 0.833, the third factor consists of 8 items with a degree of internal consistency of 0.683, and the fourth factor consists of 7 items with a degree of internal consistency of 0.703, and fifth factor consists of 5 items with a degree of internal consistency of 0.561.

Table 2 shows that the open-mindedness factor has the highest mean, followed by positive views, knowledge about research, negative views, and accessibility. The strongest correlation is between positive views and open-mindedness.

## 2 Experimental Design, Materials and Methods

The data were obtained from participants through a cross-sectional survey. The participants were registered in the faculty of teacher training and education in one of the private universities with an A (Excellent) accreditation in Jakarta, Indonesia. Samples were collected using a convenience sampling method from all students who were officially registered as students of the 8th semester or above, and they were taking undergraduate thesis research data. Three hundred fifty-two participants voluntarily filled out the questionnaire that was distributed online and offline. Their ages ranged between 20 and 24 years old, and 82.4% were males. 52.8% of the participants indicated that they had teaching experiences, while the rest had no teaching experiences. A questionnaire collected the data on teachers' beliefs about research developed by the researchers. Studies on teacher perception about research [1], [2], [3] were used to develop the questionnaire items. The questionnaire was written in Bahasa Indonesia, and then was validated by three educational experts as well as researchers. The questionnaire draft consisted of 72 items with a 6-point scale ranging from 1 (strongly disagree) to 6 (strongly agree). After the questionnaire was validated by the three experts in educational area, the eight items (i.e. 8, 16, 23, 45, 46, 50, 51, and 55) were revised according to their suggestions. Exploratory factor analysis on 72 items was performed to generate the best-fit factor structure and to reveal the best indicators for measuring each factor. Cronbach's alpha coefficient was computed to check the internal consistency of each factor. The analyses were done using SPSS version 24.

## Ethics Statement

This manuscript has not been published elsewhere or it is not under consideration for publication for other journals. The study was conducted by following Universitas Muhammadiyah Prof. Dr. HAMKA's ethical standards. Informed consent was obtained from the participants prior to the survey.

## Declaration of Competing Interest

The authors declare that they have no known competing financial interests or personal relationships which have, or could be perceived to have, influenced the work reported in this article.

## Appendix Supplementary materials

Image, application 1

## Footnotes

Supplementary material associated with this article can be found in the online version at doi:10.1016/j.dib.2020.106578.
