# Supplementary material for: The data on exploratory factor structure of [pre-service] teacher beliefs about educational research scale
Source: Data Brief. 2020 Nov 25;33:106578. doi: 10.1016/j.dib.2020.106578 (PMC7724149; doi:10.1016/j.dib.2020.106578)
Supplement: Supplementary file 1 [file mmc1.docx]

**Appendix (Original questionnaire)**

***Teachers Beliefs about Educational Research/TBER Questionnaire***

*For each statement please put (X) in the parenthesis or tick one box for each that best reflects your own understanding and practice of educational research. There is no right or wrong answer.*

| *Strongly Disagree* | *Disagree* | *Slightly* *Disagree* | *Slightly* *Agree* | *Agree* | *Strongly* Agree |
| --- | --- | --- | --- | --- | --- |
| ( ) | ( ) | ( ) | ( ) | ( ) | ( ) |

**Open-Mindedness**

1. The teacher must accept a variety of possible opinions before deciding on something.
2. Teachers must be good listeners.
3. There is no best teaching approach. *
4. Many advantages are gained when discussing problems faced in class with peers.
5. It is difficult to accept people's opinions that contradict what is believed to be true. *
6. Teachers must consider the input from others, even if it contradicts their personal opinions.
7. Teachers must be able to accept something different from what they believe is best.
8. It is inefficient tokeep changing learning approaches in class. *
9. It is important to try to implement a different approach from what has been done so far.
10. There are many alternatives that can be done to solve problems in the classroom.

**Accessibility**

1. Articles or other forms of research reports are difficult to obtain.
2. Articles or other forms of research reports are easy to access and/or find in online media.
3. Articles or other forms of research reports can be found easily in libraries or in bookstores.*
4. Articles or other forms of research reports require expensive fees to get them.
5. Communicating about educational research with experts/researchers/lecturer is easy.
6. Permission to conduct research was obtained without significant problems.*
7. Limited funds are a problem in obtaining literature.

**Value**

1. Educational research leads to teacher instruction improvement.
2. Teaching practices that are based on research results and suggestions are difficult because of time constraints.
3. Educational research can develop teacher’s knowledge for teaching.
4. Educational research results and suggestions are often not relevant to the problems encountered in class.
5. Educational research results and suggestions add alternative solutions to problems faced in class.
6. Educational research results does not have a significant impact on teaching practice.
7. Educational research helps improve student learning.
8. Educational research is needed to formulate educational policies.
9. Educational research is often out of sync with the policies implemented.
10. Educational research provides time for self-reflection on the teaching undertaken.*
11. Educational research is only useful for administrative purposes (graduation; rank).
12. Educational Research develops logical thinking ability.
13. Educational research trains teachers to adapt to various learning conditions and situations.
14. Educational research suggestions are difficult to apply in the form of real practice.
15. Educational research is needed to evaluate what has been taught, to what extent, and what is needed next.
16. Educational research is needed as a solution to overcome educational problems.
17. Educational research results have theoretical and/or practical values.
18. Educational research results are not convincing enough to be recognized for accuracy.
19. Educational research develops the ability to make careful planning.
20. Educational research is a matter of researchers’ personal interest.
21. Educational research develops the ability of teachers to manage classrooms and interact with students.
22. Learning theories are often not relevant to the reality of the educational settings.
23. Educational research motivates oneself to try different approaches to classroom practice.*
24. There is no problem even though there is no research.
25. Educational research helps to recognize student characteristics.

**Engagement**

1. Teachers need to do research for each material taught. *
2. Teachers will not have enough time to manage between teaching and learning activities and research activities.
3. Each teaching should be based on research results and suggestions. *
4. A teacher is a researcher. *
5. Research takes time away from other teacher’s responsibilities.
6. A teacher conducting research is a form of self-actualization. *
7. My friends and I often wonder why I have to do research.
8. Educational research was carried out because of administrative encouragement (graduation; rank).
9. It is important to actively communicate ideas or research results in various media and/or forums. *
10. I have not felt the impact of research on the educational field until now.
11. Being active in workshops or seminars or similar forms can contribute ideas to research. *
12. Research is carried out because of encouragement from rules and policies.
13. It is important to collaborate with researchers / lecturers / experts to conduct research. *
14. Peer-Discussion helps to improve the quality of the research being carried out. *

**Knowledge about research**

1. Research must be in accordance with the template (form, format, or pattern) of previous studies. *
2. A good title is able to reflect the contents of the research.
3. Good research is one that can be completed quickly. *
4. It is important to publish educational research results.
5. Research and publications complement each other.
6. The research method depends on the research purpose.
7. Research results can be directed according to the needs of the researcher. *
8. Problem statements can be derived from a review of previous research results.
9. The research focus must be adjusted to the prevailing policies. *
10. Research is conducted to test the theories that have been formulated.
11. Literature review helps to conclude while the results of research to be carried out.*
12. Good references take precedence over research results.
13. Textbooks are the main source of reference in conducting research. *
14. Research results must be used as references more than textbooks.
15. References should be avoided on the background of the problem. *
16. Personal experiences can be the background of problems. *

***Items deleted**
